# Activity and Mechanism of Action of Antifungal Peptides from Microorganisms: A Review

**DOI:** 10.3390/molecules26113438

**Published:** 2021-06-05

**Authors:** Tianxi Li, Lulu Li, Fangyuan Du, Lei Sun, Jichao Shi, Miao Long, Zeliang Chen

**Affiliations:** 1 College of Animal Science & Veterinary Medicine, Shenyang Agricultural University, Shenyang 110866, China; 2020240619@stu.syau.edu.cn (T.L.); lilulu981121@163.com (L.L.); fangyuandu@126.com (F.D.); 2 College of Animal Husbandry and Veterinary Medicine, Jinzhou Medical University, Jinzhou 121001, China; jzyxysl@163.com; 3 Liaoning Agricultural Development Service Center, Shenyang 110032, China; sjc6319@126.com

**Keywords:** antifungal peptide, antibacterial activity, antibacterial stability, cytotoxicity, antifungal mechanism

## Abstract

Harmful fungi in nature not only cause diseases in plants, but also fungal infection and poisoning when people and animals eat food derived from crops contaminated with them. Unfortunately, such fungi are becoming increasingly more resistant to traditional synthetic antifungal drugs, which can make prevention and control work increasingly more difficult to achieve. This means they are potentially very harmful to human health and lifestyle. Antifungal peptides are natural substances produced by organisms to defend themselves against harmful fungi. As a result, they have become an important research object to help deal with harmful fungi and overcome their drug resistance. Moreover, they are expected to be developed into new therapeutic drugs against drug-resistant fungi in clinical application. This review focuses on antifungal peptides that have been isolated from bacteria, fungi, and other microorganisms to date. Their antifungal activity and factors affecting it are outlined in terms of their antibacterial spectra and effects. The toxic effects of the antifungal peptides and their common solutions are mentioned. The mechanisms of action of the antifungal peptides are described according to their action pathways. The work provides a useful reference for further clinical research and the development of safe antifungal drugs that have high efficiencies and broad application spectra.

## 1. Introduction

Fungi are eukaryotic microorganisms that exist widely in nature and include molds, yeasts, and so on. Many of them, e.g., Fusarium graminearum, Aspergillus ochraceus, A. flavus, are a great threat to the normal growth of crops, foodstuffs, and feed safety. When mycotoxins are ingested to a certain level by humans and livestock, they cause serious adverse reactions, such as, fungal infection, hepatorenal toxicity, carcinogenesis, and teratogenesis [1].

Every year, huge economic losses are incurred in the process of growing, storing, and transporting crops due to mildew. Such losses have a great impact on our food production industry and our lives. Unfortunately, most of the antifungal drugs that can be used in the treatment of fungal diseases are polyenes, triazoles, echinocandins, and some other auxiliary drugs (e.g., 5-fluorocytosine), the application of which can be toxic to patients to different degrees. Worse still, more and more drug-resistant strains are emerging [2,3], and the drug resistance of some fungi is also rising year-by-year [4,5]. This suggests there is an urgent need to develop new antifungal drugs [6].

Antimicrobial peptides are small molecules produced by organisms that play an important role in the innate immunity of the organism [7] (the name was first formally proposed by the Swedish scientist G. Boman in 1981 [8]). These antimicrobial peptides are not only active against pathogenic microorganisms directly but also play a role in regulating the autoimmune system of the host. Thus, they have wide application prospects in the prevention and treatment of animal- and plant-related diseases, the development of new drugs, and the field of biological detoxification [9].

Antifungal peptides are antimicrobial peptides with antifungal activity. Scientists isolated antifungal substances from Bacillus subtilis as long ago as 1948 [10]. Since then, some studies on the mode of action and biosynthesis of antifungal antibiotics began to appear [11,12]. Boman et al. also found substances with antifungal effects in their continued studies of antimicrobial peptides that they had previously discovered [13]. To date, there are about 2700 kinds of antimicrobial peptides in the Antimicrobial Peptide Database, but only 1000 or so have antifungal activity. At present, many antifungal peptides are found and defined as part of studies generally aimed at antimicrobial peptides. However, targeted research on antifungal peptides is developing rapidly, and there are many kinds of antifungal peptides that have been isolated and purified from microorganisms.

In this review, the antifungal peptides available from microbial sources are summarized and discussed according to different classification methods. The aim is to produce a reference work that will be helpful for those conducting further research in this field in the future.

## 2. Microorganisms Producing Antifungal Peptides

There are three kinds of microorganism that produce antifungal peptides: bacteria, fungi, and actinomycetes. Of these, bacteria form the largest group of sources, of which *Bacillus subtilis*, *B. amyloliquefaciens*, *B. cereus*, and so on are widely used in research on biological control.

Li et al. extracted and analyzed an antifungal substance from a strain of *B. amyloliquefaciens* sybc H47 and found that it had a significant effect on a variety of pathogenic fungi, e.g., *Aspergillus niger*, *Fusarium oxysporum*, *Penicillium citrinum*, and *Candida albicans* [14]. Other of bacterial species can also produce antifungal peptides, but the number of papers on them is far less than those related to *Bacillus* species (at present).

Many fungi have been found to synthesize antifungal peptides. For example, *Aspergillus* can synthesize echinocandins that are resistant to invasive fungal infections [15].

With the rapid development of antifungal peptide research, people are not satisfied with the discovery of new antifungal active substances only from terrestrial microorganisms. As a result, researchers are beginning to focus on ocean sources. For example, the excellent antifungal activity of marine actinomycetes has attracted more and more attention from researchers in recent years [16]. *Streptomyces* species have been widely studied and found to have many other functions in addition to antifungal activity. For example, they also have antibacterial, antiviral, and antiparasitic properties, etc., which makes them very valuable in the development of new antibiotics [17].

Table 1 presents a summary of the antifungal microorganisms of different species found to date as well as the species of fungi they act upon. It can be seen that most of the antifungal peptides have broad-spectrum antifungal effects (i.e., they can act on a variety of pathogenic fungi of the same or different species of relevance to plants and animals). Researchers are also working to expand the antibacterial spectra of antifungal substances that act upon single antifungal species by changing the structures of the compounds involved. For example, naturally occurring echinocandins and their semisynthetic derivatives are limited by their narrow antibacterial spectra. For instance, the activity spectrum of anidulafungin only includes *Candida* and *Aspergillus* infections—it has no activity against *Cryptococcus* spp., *Fusarium* spp., or *Trichosporon* spp. [18]. On the basis of the existing structure–activity relationship, researchers subsequently synthesized a variety of cyclohexalipopeptide compounds. The activity test results show that some of these synthesized compounds not only have stronger anti-*Candida albicans* activity than caspofungin but also feature in vitro inhibitory activity against *Cryptococcus neoformans*, *Aspergillus fumigatus*, and *Trichoderma rubrum* [19].

Structural modification may also help improve the solubility and stability of such drugs in addition to expanding their antibacterial spectra [20]. Moreover (as can be seen from Table 1), the antifungal targets of many antifungal peptides from different sources overlap. This means that combinations of drugs can be used that will help reduce drug resistance. The antibacterial effects of many antifungal peptides are very significant [15], and therefore their minimum inhibitory concentrations (MICs) are correspondingly low [21]. For example, the MIC value of the antifungal peptide P-1 with respect to *Trichothecium roseum* has been found to be as low as 1 µg/mL [22]. This is highly significant when it comes to improving the therapeutic effect of an antibacterial drug while reducing the dosage required.

**Table 1:** Antifungal microorganisms derived from different species and genera and the species of fungi they act upon.

Microbial Species	Source	Name of Antifungal Peptide	Molecular Weight/Da	Fungal Species	References
*Bacillus* BH072	*Bacillus*	Flagellin	35 615	*Aspergillus niger, Pythium, Botrytis cinerea, Fusarium oxysporum*	[23]
*Bacillus* AH-E-1	*Bacillus*	Not named	500-1000	A variety of plant and human pathogenic fungi	[24]
*Bacillus* B9987	*Bacillus*	Metabolites BMME-1	Not mentioned	*Alternaria solani*	[25]
*Bacillus subtilis* CCTCCM207209	*Bacillus*	Iturin A	1095.5	*Candida, Hyphomyces cerevisiae, Fusarium* and *Aspergillus*	[26,27]
*Bacillus subtilis* B25	*Bacillus*	Not named	38708.67	*Fusarium oxysporum, Alternaria solani, Corynespora, Botrytis cinerea, Colletotrichum gloeosporioide*	[28]
*Bacillus amyloliquefaciens* SWB16	*Bacillus*	Subtilin, Iturin	1042.6-1505.9	*Beauveria bassiana*	[29]
*Bacillus cereus* YQ 308	*Bacillus*	Chitinase, chitosanase, protease	48,000	*Fusarium oxysporum, Fusarium solani, Pythium ultimum*	[30,31]
*Bacillus thuringiensis* S4	*Bacillus*	Chitin-binding protein CBP24	21,000	*Fusarium, Rhizoctonia subtilis*	[32]
*Bacillus licheniformis* W10	*Bacillus*	Serine protease	48,794.16	*Botrytis cinerea*	[33]
*Bacillus pumilus* HN-10	*Bacillus*	P-1	1149.14	*Trichothecium roseum*	[22]
*Bacillus bereis* DTU001	*Bacillus*	Not named	Not mentioned	*Candida, Penicillium, Aspergillus,* etc.	[34]
*Paenibacillus polymyxa* KT-8	*Paenibacillus*	Fusaricidin A	About 883	*Fusarium oxysporum, Aspergillus niger, Saccharomyces cerevisiae, Magnaporthe grisea,* etc.	[35,36]
*Paenibacillus ehimensis* MA2012	*Paenibacillus*	Not named	1115	A variety of plant pathogenic fungi, *Colletotrichum*	[37]
*Pseudomonas syringae*	*Pseudomonas*	Syringostatin A, syringostatin E	About 1179.7, About 1161.3	*Yeasts, filamentous fungi*	[21]
*Helicobacter pylori*	*Spirillum*	HP 2-20	About 2320.8	*Candida albicans, Hyphomyces burnetii*	[38]
*Enterococcus faecalis*	*Enterococcus*	EntV	3000-10,000	*C. albicans, C. tropicalis, C. paraplanatus,* etc.	[39]
*Aspergillus nidulans*	*Aspergillus*	Echinocandin B	About 1 060.2	*Candida*	[15]
*Aspergillus clavatus*	*Aspergillus*	AcAFP	5773	*Fusarium oxysporum, Aspergillus niger, Botrytis cinerea,* etc.	[40]
*Penicillium citrinum* W1	*Penicillium*	PcPAF	About 10,000	*Trichoderma viride, Fusarium oxysporum, Paecilomyces variotii, and Alternaria longipes*	[41]
*Aureobasidium pullulans*	*Aureobasidium*	Aureobasidin A(AbA)	1070-1148	*Candida, Cryptococcus neoformans, Blastomyces dermatitis,* etc.	[42,43]
*Acremonium persicinum*	*Acremonium*	VL-2397	About 914.9	*Aspergillus, Cryptococcus neoformans, Candida glabrata,* etc.	[44]
*Marine streptomyces* DA11	*Streptomyces*	Chitinase	About 34000	*Aspergillus niger, Candida albicans*	[45]
*Marine Actinomycetes* M045	*cladothrix actinomyces*	Chandrananimycin A	About 270.24	*M. miehei*	[46]
*Actinomycete Streptomyces cacaoi*	*Streptomyces*	Polyoxin D	About 521.4	*Candida albicans, Cryptococcus neoformans,* etc.	[47]
*Streptomyces tendae*	*Streptomyces*	Nikkomycin Z	About 495.4	*Glomus, Aspergillus fumigatus,* etc.	[48,49]

## 3. Stability of Antifungal Peptides

In general, the physical and chemical properties of the antifungal substances extracted from microbial fermentation broths (via separation and purification) need to be determined so that their stability can be ascertained and their suitability for antibacterial application decided. This mainly involves determining their structural stability to acidic/alkaline conditions and heat as well as with respect to various proteases, metal ions, chemical reagents, and ultraviolet (UV) light. Naturally, it is very helpful to ascertain the category to which the antifungal substance belongs (lipopeptide, polypeptide, protein, etc.). The properties of the substance thus determined clearly dictate the conditions required for its industrial production.

To determine the tolerance of the substance to acidic/alkaline environments, we need to ascertain the pH range over which the activity of the antifungal peptide is relatively stable. This range may be around neutral conditions [23], but there are also some results biased to acidic [50]/alkaline [51] conditions. Moreover, the highest antifungal activity is not always manifested in neutral solution. For example, the antifungal peptide PcPAF mentioned in Table 1 is most active in weakly acidic environments [41].

The highest temperature tolerated by most antifungal peptides corresponds to 100 ℃ [52,53]. However, Zhao et al. found active metabolites (produced by endophytic *Bacillus vallismortis* ZZ185) that could maintain over 50% of their antifungal activity after exposure to 121 °C for 30 min [54].

Surfactants (e.g., sodium dodecyl sulfate and urea), organic compounds (e.g., ethylenediaminetetraacetic acid, trichloroacetic acid, chloroform), and ammonium sulfate are commonly used to test the stability of antifungal peptides. Researchers usually focus on antifungal peptides that are not sensitive to these chemicals [55]. Moreover, some active substances will show higher activity and stability in the presence of surfactants [56]. Many other chemical species are also commonly used when separating and purifying antifungal peptides and thus we also need to make sure the separation method chosen does not affect the activity of the antifungal component.

To test an antifungal substance for UV stability, the substance is irradiated with UV for different times and at different doses [57]. As for protease stability, the anti-degradation effect of the antifungal peptide on protease K, papain, pepsin, trypsin, etc. is often determined. The sensitivity of the different antifungal peptides to these substances is different, which is the main index used to judge the category to which the antifungal peptide belongs [22,28,58].

Metal ions (K^+^, Na^+^, Mg^2+^, Ca^2+^, Zn^2+^, Cu^2+^, etc.) also affect the activity of some antifungal peptides. Different ions have different effects on the same antifungal peptide, and the effect of the same ion on different antifungal peptides is not necessarily the same [53,59]. This reflects the different interactions that occur between the ions, fungi, and antimicrobial peptides, and such investigations can be of help when inferring the mechanism by which the antifungal peptide functions.

## 4. Toxicity of Antifungal Peptides

Once an antifungal species has been identified and its activity stability determined, its potential for further research and clinical application depends on its toxicity to the body. After all, one of the main disadvantages of some existing antifungal drugs is that they are highly toxic to the liver, kidneys, and/or blood. For example, the drugs traditionally used to treat deep invasive fungi (azole or polyene drugs) can lead to hepatotoxicity [60] or nephrotoxicity [61], causing extensive damage to different parts of the body under the action of multiple factors.

Echinocandins, e.g., caspofungin (a kind of lipopeptide antifungal substance) [62], on the other hand, are relatively safe to use in mammals because of their unique antifungal mechanism (acting as they do on the walls of the fungal cells). Compared with other antifungal drugs, these produce mild adverse reactions, including local phlebitis, fever, liver dysfunction, and mild hemolysis [63]. Moreover, these adverse reactions have been gradually reduced in severity as these antifungal drugs have continued to be updated and improved [64].

Currently, the main adverse effect produced by certain antifungal peptides is hematotoxicity represented by erythrocyte hemolysis, which occurs to different degrees of severity. For example, pulmonary *Candida albicans* A0 has a high degree of hemolytic activity. Moreover, although it has effective fungicidal activity against *Candida albicans*, it lacks efficacy against some *Aspergillus* and other *Candida* species. Therefore, the practical application of pulmonary *Candida albicans* A0 needs to be given careful consideration [65].

It has also been reported that syringostatins A and E (isolated from *Pseudomonas syringae*) and iturin A (isolated from *Bacillus subtilis*) exhibit erythrotoxicity [21,66]. At the same time, the antifungal active lipopeptide extracted from *Bacillus amyloliquefaciens* SWB16 also has the *iturin A* gene, and thus it is possible that this antifungal substance will also exhibit erythrotoxicity [29]. Of course, some antifungal peptides (e.g., peptide Cm-p5) [67] and broad-spectrum antibacterial substances (e.g., AbA, residue 2–20 from *Helicobacter pylori* ribosomal protein and its analogues HPA3) [68,69] with low toxicity to mammals have been found.

In addition to hemolysis, antifungal peptides can also cause damage to DNA, e.g., actinomycin D (a member of the chromopeptide family) [70]. Although this substance has a significant effect on Verticillium wilt (which is caused by a fungal infection) [71], it can induce extensive and rapid apoptosis as it is widely recognized to be an inhibitor of RNA synthesis [72].

In this context, reduction or elimination of the toxicity of antifungal substances has long been an important issue. In addition to developing new varieties of antifungal peptides in order to find compounds with low toxicities, increasing numbers of researchers are focusing on structurally modifying existing antifungal substances or adding drug-loading systems to remove or hide their original toxicity. For example, echinocandin B is highly toxic to the blood of mammals as a result of its hemolytic behavior. In response, scientists have developed a semisynthetic analogue, cilofungin, which is significantly less toxic than echinocandin B [73]. The use of liposomes as a drug delivery system has also been found to perform well in anti-infection studies [74]. For example, when amphotericin B is administered as a liposomal formulation, its nephrotoxicity is significantly reduced, even though there is no significant change in its efficacy [61]. In addition, external solutions can be added to prevent hemolysis through appropriate osmotic protectants [66].

## 5. Mechanism of Action of Antifungal Peptides

Generally speaking, the mechanism by which antifungal peptides function is either to inhibit the growth and reproduction of the pathogen or to directly kill it. Depending on their target, they can be divided into three broad categories in which their action is aimed at: the pathogenic fungi, their own strains, or the cells of the host itself. It is also possible that new targets will be found for the drugs.

Knowledge of the mechanisms combined with the results of an analysis of the structure of the antifungal peptide provides a useful reference for the synthesis of new antifungal compounds with stronger antifungal activity, broader antibacterial spectra, and lower toxicity to the host. At present, however, our knowledge of the mechanisms by which antifungal peptides function is not complete. The following summarizes some of the widely recognized mechanisms by which antifungal peptides work.

### 5.1. Effect of Antifungal Peptides on Pathogenic Bacteria

From a macroscopic point of view, antifungal peptides inhibit or kill pathogenic fungi by inhibiting mycelial growth; affecting spore germination; or causing the hyphae or spores to become broken, swollen, twisted, or deformed, etc. Generally speaking, antifungal peptides have different effects on the survivability of pathogenic fungi. However, there are also antifungal peptides that only affect the morphology of the mycelium [39]. *Bacillus* AH-E-1 can distort the hyphae of *Candida albicans* and other fungi, and the antifungal substances extracted from its fermentation supernatant can inhibit spore germination, germ tubes, and hyphal growth of filamentous fungi [24]. The antifungal peptide EP-2 produced by *Bacillus subtilis* E1R-J can swell and distort the mycelium of the fungi that causes apple canker, leading to the exosmosis of protoplasts, thus inhibiting the growth of the fungi [53]. The specific targets involved can be described in terms of the structure of the cells.

#### 5.1.1. Targeting of Cell Walls

Antimicrobial peptides act on microbial cell walls via a characteristic mechanism that is different from that encountered using other antifungal drugs. This effectively avoids the problem of high toxicity to mammalian cells. The mechanism by which antifungal and antimicrobial peptides act on cell walls is different mainly because of the different components in their cell walls (mainly glycans) [75]. The cell walls of fungi are composed of carbohydrates (e.g., glucan), chitin, glycoproteins (e.g., mannoproteins), and various proteins [76]. It has been confirmed that some antifungal peptides can affect the synthesis of these main components, thus causing damage to the cell walls.

β-Glucans are the main polysaccharides in the cell walls of fungi. They have a network structure formed by connecting glucose monomers via β-(1,3)- or β-(1,6)-glycoside bonds. The network structure produced has a supporting effect on the cell walls and a variety of specific receptor sites on its surface are of great significance as they help the fungi to recognize and induce host immune responses [77]. Some antifungal peptides, e.g., echinocandins, are non-competitive inhibitors of β-(1,3)-glucan synthase, which affects the synthesis of fungal cell walls. This is the main way in which caspofungin [62], micafungin [78], and anifgin function. More specifically, the semisynthetic lipopeptide anifgin expresses its antibacterial activity by inhibiting the synthesis of (1,3)-β-d-glucan in the cell walls of *Candida* and *Aspergillus*, and this allows it to have an inhibitory effect on strains that are resistant to azole or polyene antifungal drugs [18]. In addition, studies have shown that some other compounds of the echinocandin family also function via the same mechanism of action [65,79]. The development and optimization of these compounds are thus promoting the development of new synthetic antifungal drugs for clinical use.

Chitin is an amino polysaccharide composed of *N*-acetyl-d-glucosamine units [80]. It is an important component of fungal cell walls and can therefore affect the regulation of cell viability and host immune response [81]. It has been found that changing the chitin content directly affects the sensitivity of some fungi to antifungal agents [82]. However, it cannot help the host to escape its fate completely. It has also been reported that although fungal chitin can induce and activate a variety of plant defense responses, the fungi can convert chitin into chitosan in the process of plant infection in order to escape the plant defense mechanisms [83]. Many antifungal substances extracted from *Streptomyces* species act on chitin. For example, Mizuhara et al. isolated cyclothiazomycin B1 from *Streptomyces* HA 125-40 that causes cell walls to rupture by binding with the chitin, leading to the death of fungal cells [84]. Other examples are nikkomycin and polyoxin, which are competitive inhibitors of chitin synthase [85,86] and have effect on many kinds of pathogenic bacteria [47,48,87]. A combination of nikkomycin Z and echinocandins has also been found to produce a synergistic effect against *Aspergillus fumigatus* [49], which is also related to their effect on chitin [82,88]. In addition to *Streptomyces* sources, the antifungal cyclic lipopeptide, chromobactomycin, which was obtained by Kim et al. from *Chromobacterium* C61 has also proved to be the key to the antibacterial effect of C61 according to in vitro experiments (by adding chitin to the medium) [89].

Mannan is found in the outermost layer of fungal cell walls. It can be glycosylated with proteins or peptides of different adhesion properties to form mannoprotein macromolecules. It has strong adhesive properties and determines the adhesion of the fungi to the host cells [90]. In addition, mannan also plays an important role in immune recognition and virulence of fungi [91]. The activity of the antifungal compound pradimicin (PRM) is aimed at cell wall mannan. Studies have shown that PRM can specifically recognize and bind to the D-mannoside sites in the cell walls of *Candida albicans* forming a ternary complex of pradimicin, D-mannoside, and calcium, thus destroying the integrity of the fungal cell membrane and achieving an antifungal effect [92]. This mechanism endows it with broad-spectrum antifungal activity in vitro against *Candida* species, *Cryptococcus neoformans*, *Aspergillus* species, dematiaceous molds, etc. It also has no major end-organ toxicity and a good therapeutic index. It is the basis of a new class of antifungal compounds that are in preclinical and early, phase I clinical trials [92]. PRM can also induce cell apoptosis in *Saccharomyces cerevisiae* through the accumulation of reactive oxygen species [93] and it can also act on the *N*-glycosylation site of the osmotic-sensitive protein Sln1 and thus play a bactericidal role [94]. Benanomicin and other members of the same family have also been found to function via a similar antifungal mechanism (binding cell wall mannan sites) [95].

Microorganisms can also remove pathogenic fungi via physical adsorption. For example, polysaccharides, proteins, and lipids on the surfaces of cell walls can adsorb mycotoxins through hydrogen bonds, ionic bonds, and hydrophobic interactions, thus achieving detoxification [96]. Bejaoui et al. [97] found that *Saccharomyces cerevisiae* and *S. bayanus* yeasts can remove ochratoxin A from grape juice by physical adsorption. Furthermore, dead yeast cells gave a better adsorption effect than living yeast cells, which means the former can be used as a safe and effective method of biological detoxification.

#### 5.1.2. Targeting Cell Membranes

To date, most of the antimicrobial peptides found in nature target cell membranes in order to exert their effects and a wide range of activities are involved. The realization of this action depends mainly on the physicochemical properties of antifungal peptides and target membrane tissues, which are the determinants of stable peptide–membrane interaction. For example, the electrostatic bonding between the antibacterial peptide and the surface structure of the target membrane due to the opposite charge will attract the antibacterial peptide to the cell membrane. Later, due to the amphiphilic nature of the antimicrobial peptides, they can combine with the lipid bilayer to form different secondary structures (such as α-helices, β-sheets, and so on). This is essential for the expression of their antibacterial activity. Only those peptides that can form highly amphiphilic structures have significant antibacterial activity [98], and the selectivity of different conformations to lipids is also different [99]. Moreover, Martins et al. discovered through the study on Trialysin that the selectivity of the active peptides for specific organisms appears to be associated with the structural features of their N- and C-termini [100]. In addition to these linear configurations, antibacterial peptides also commonly exhibit a circular conformation, forming a helix II structure, and intramolecular disulfide bonds play an important role in stabilizing the configuration [101]. In addition, hydrophilicity and hydrophobicity are also important properties of many antimicrobial peptides, which make antimicrobial peptides not only soluble in aqueous environment but also enter lipid rich membrane [102]. This property is closely related to the cytotoxicity and selectivity of antimicrobial peptides [103]. Researchers often modify natural antimicrobial peptides by hydroxylation, glycosylation, lipidization, and cyclization in order to optimize their properties (such as improving their stability and bioactivity) [104].

The research shows that there are two different physical models for the binding of antimicrobial peptides to lipid bilayers. The difference between them is the ratio of peptide to lipid (P/L) [105], which determines the sensitivity of cells to antimicrobial peptides. At low P/L, antimicrobial peptides tend to adsorb and embed into the lipid head base region in a state of functional inactivation and bind with lipid bilayers in parallel. With the increase of P/L ratio, the peptide began to act vertically on the membrane until it was inserted into the lipid bilayer to form a transmembrane pore (called state I) [105]. The ratio of type I peptide to lipid varies with the composition of peptide and target lipid, which can be described by three different action models [106]. 

*Barrel wall model*—In this model, helical peptides aggregate within the wall and form fascicular pores in the membrane with a central cavity, thus affecting the permeability of the membrane [107]. Amphotericin B (AMB), a polyene antifungal agent, is the only natural product that produces an antifungal effect via this mechanism [108]. AMB targets membrane sterol, its antifungal activity reflected in the formation of transmembrane ion-permeable self-assemblies with ergosterol [109,110]. This mechanism provides a reasonable explanation for the selective toxicity of AMB [111]. This particular mode of intermolecular interaction has been verified in many experiments involving phospholipid monolayers [112] and bilayers [113], but the specific interaction mechanism needs to be studied further. It has also been found that fluconazole can reduce ergosterol content, and its combination with AMB can produce an antagonistic effect [114]. Interestingly, the glycotriazole peptides prepared by Junior et al. showed similar effects to fluconazole. The fungicidal activity of these peptides can be demonstrated by inhibiting ergosterol biosynthesis, which seems to be related to the presence of both the monosaccharide and the triazole rings [115].

*Carpet-like model*—In this model, the peptide covers the membrane surface in a carpet-like manner and interact with the membrane in parallel due to the electrostatic interaction with the anionic phospholipid head group. The formation of micelles at high peptide concentration destroys the phospholipid bilayer [116]. This mechanism makes lysate peptides can lyse cells of different microorganisms and normal mammalian cells [116], causing obvious toxicity problems. The syringomycin family of lipopeptides secreted by *Pseudomonas syringae* belongs to this group. The transmembrane pores formed by these lipopeptides are permeable to cations and cause pathogen necrosis. The main reason for hemolysis is that it forms ion channels in the cell membrane and makes the colloid dissolve [117]. However, it has also been reported that the presence of cholesterol can reduce the binding of antimicrobial peptides to various lipid bilayer model membrane systems, thereby decreasing the lytic capacity of these peptide on the eukaryotic cells [118,119].

*Annular pore model*—In this model, a peptide helix is first inserted into the membrane to form a pore and the lipid monolayer is continuously bent until it passes through the membrane. The hydrophilic structure of the membrane then wraps the two sides to together forming a toroidal-shaped pore hole [120]. This mode of action has been widely verified in a variety of animal-derived antifungal peptides, such as melittin (found in bee venom) [121] and Xenopus antimicrobial peptide (found in *Xenopus* skin) [122].

There are many other antifungal peptides that exert their activity by interacting with membranes via mechanisms that are not yet fully understood. The lipopeptide iturin produced by *Bacillus* species can interact with target cell membranes to form ion pores in the membrane, thus increasing the permeability of the pathogen cell membrane to potassium ions. The effective structure produced may be a ternary structure of the form iturin/phospholipid/sterol [123]. Actinomycin D can fold and split the plasma membranes of pathogenic fungi, destroying the membrane and leading to leakage of the cell contents [71].

Sphingolipids are also found in the plasma membranes of eukaryotic cells and are known to play important roles in cell growth, apoptosis, signal transduction, etc. [124]. The synthesis of inositol phosphorylceramide (IPC) is a key step in the synthesis of sphingolipids in fungi. It has been shown that the cyclic non-ribosomal peptide aureobasidin that is produced by *Aureobasidium pullulans* can inhibit sphingolipid synthesis by noncompetitive inhibition of IPC synthase, thus generating antibacterial action against *Candida* species and *Cryptococcus neoformans* [125]. Due to the lack of target enzymes in mammalian cells, it has also become a potential target for the development of non-toxic antifungal drugs. This has been verified in activity tests on IPC-synthase deficient mutants. The death of the mutant cells is accompanied by the accumulation of ceramide, suggesting that the existence of the ceramide activates the death response [126].

#### 5.1.3. Targeting Nucleic Acids, Organelles, and Intracellular Macromolecules

The effects of antifungal peptides (on pathogenic fungi) are not limited to the destruction of the wall membranes. Rather, they can also affect the nuclei, organelles, and intracellular proteins after entering the cells. Using various analytical methods (LC-HRMS analysis, Student’s *t*-test, etc.), Aspasia katragkou et al. [127] discovered a new mechanism of action of micafungin: by inhibiting the protein synthesis and cell replication of pathogenic fungi, it induces changes in their metabolic pathways.

Lee et al. studied the antifungal mechanism responsible for the action of a 14 helix β-peptide [128]. They found that after the β-peptide enters the cytoplasm (by interacting with the plasma membranes to form pores), it destroys the nucleus and vacuole, in turn leading to cell death. Through proteomic analysis and a series of validation tests, Han et al. determined the action pathway of the cyclic lipopeptide AMP-jsa9 (produced by *Paenibacillus polymyxa* jsa-9) against Fusarium moniliforme [129]. The lipopeptide not only targets the cell membrane structure and enhances the leakage of potassium ions, proteins, and other components of the cytoplasm, but also regulates the levels of various intracellular proteins. It can thus strongly affect the normal life activities of the cells, affecting their structure and metabolism.

In addition to the above mechanisms, the special structures of some antifungal peptides also determine their unique antibacterial pathways. For example, a new type of *Aspergillus*-resistant, aluminum-chelating, cyclic hexapeptide VL-2397 (formerly known as ASP2397) has recently attracted much attention [130]. Its structure is similar to that of a ferrichrome-type siderophore (which can absorb iron from the outside through the iron carrier transporter on the cell membrane). The xylose-dependent iron carrier transporter gene *sit1* occurs in the plasma membrane of some *Aspergillus* species, e.g., *A. fumigatus* and *A. flavus*, and the expression of this gene determines the uptake of VL-2397 by the *Aspergillus* species [130]. The interaction between VL-2397 and intracellular targets inhibits mycelial elongation, thus achieving an antibacterial effect. As mammalian cells do not contain the corresponding genes, the antifungal peptide has good prospects for use in clinical applications [131].

### 5.2. Effects of Antifungal Peptides on Their Own Strains

Antifungal peptides can also affect cluster movement and the colonization and biofilm formation ability of some bacterial strains. They can thus promote the mass propagation or colonization of bacteria in plant roots, which can inhibit the growth of pathogenic fungi and thus protect the plant [132]. Experiments have shown that bacillomycin D produced by the rhizosphere strain *Bacillus amyloliquefaciens* SQR9 plays a vital role in the antagonistic activity of SQR9 against *Fusarium oxysporum* [133]. This was confirmed by testing the antagonistic activity of mutant species lacking this lipopeptide. In addition, the bacillomycin D was found to improve the expression level of the *kinC* gene, which promotes biofilm formation and the growth and colonization of the SQR9 itself, helping to prevent the *F. oxysporum* from causing plant wilt.

### 5.3. Competitive Effects of Antifungal Peptides on Host Targets and Nutrients

In addition to antibacterial activity, antimicrobial peptides have been found that can interact directly with host cells by modulating the inflammatory and innate defense mechanisms [134]. For example, through regulation of the expression of plant proteins and metabolism level, it is possible to strengthen the plant’s defense system, promoting growth and inducing positive changes in its disease resistance and physiological function [135,136,137]. In this way, *Trichoderma* strains can interact with olive leaf spot and the pathogenic fungi of olive leaf spot to produce secondary metabolites, induce the expression of defense-related genes, and enhance the disease resistance of the plants [138].

At the same time, many growth-promoting bacteria occupy the same action sites on plants as pathogenic fungi. Therefore, both compete for the nutrients secreted by the plants and already present in the environment. This can be used to inhibit, or even eliminate, the pathogenic fungi, an approach that has been widely used in plant disease control [139]. *Metschnikowia citriensis* strain FL01 exerts a biocontrol effect on citrus sour rot in citrus fruit by rapidly colonizing wounds on the fruit and competing for nutrition and space with the pathogenic fungi [140]. It promotes biofilm formation in the citrus fruit and inhibits mycelium growth and spore germination. However, it cannot produce cell metabolites or volatile organic compounds that have antibacterial effects. *Cryptococcus laurentii*, on the other hand, also has an antagonistic ability towards this pathogen that can be attributed to its competing for the same nutrients and space, its defense responses, and its ability to secret antibiotics [141].

### 5.4. Brief Summary

**Figure 1:**
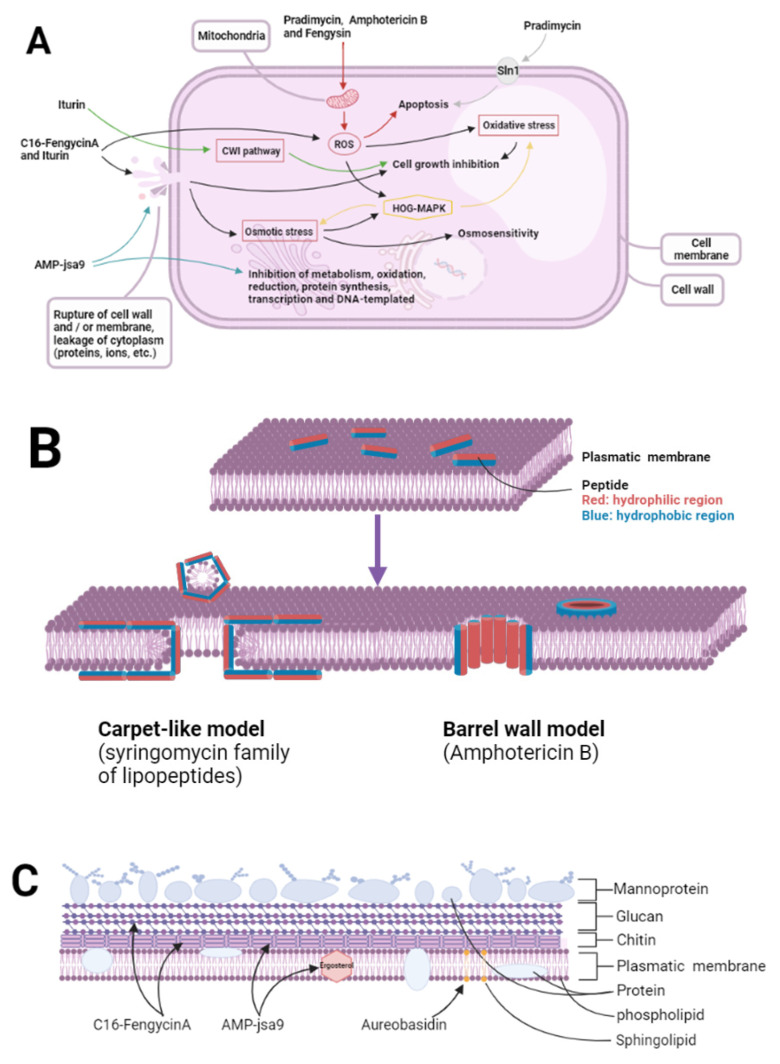
Action pathways and mechanisms of action of some antifungal peptides. (**A**) the mechanism of some antifungal peptides; (**B**) two models of destruction of cell membrane by mi-crobial antifungal peptides; (**C**) the main membrane structure of pathogenic fungi affected by antimicrobial peptides.

Figure 1 summarizes the mechanism of some antifungal peptides mentioned above. There are several antimicrobial pathways, such as oxidative stress, osmotic stress, apoptosis, destruction of cytoskeleton structure, and cell metabolism disorder. It is not difficult to see that most antifungal peptides rely on a single antibacterial mechanism. However, they often act on a variety of structures and combine a variety of ways to achieve their antibacterial purpose. For example, iturin can induce oxidative stress through the accumulation of reactive oxygen species and can interact with target cell membranes to regulate protein levels, which can lead to cell wall rupture. Moreover, cytoplasmic extravasation caused by cell rupture can also induce cell osmotic stress. In addition, it can also act as an activator to induce plant defense response to pathogenic fungi [142]. These pathways play an important role in the antibacterial activity of iturin. However, the accumulation of reactive oxygen species and osmotic stress also activate the HOG-MAPK pathway, which can resist the damage of oxidative stress and osmotic stress. This pathway may be related to fungal drug resistance [142].

The effects of fengycin on some *Candida* and *Rhizopus* species are also manifested in many ways, such as the destruction of pathogen cell walls; inhibition of DNA synthesis; and apoptosis marked by accumulation of reactive oxygen species, mitochondrial dysfunction, and phosphatidylserine eversion [143,144]. Some *Bacillus* species produce lipopeptides called surfactins that are highly potent biosurfactants that have similar anti-*Candida* and anti-*Fusarium moniliforme* activities to fengycin [145]. The combined effect of surfactin and fengycin has been studied but the results achieved were not ideal [146]. However, combination therapy, especially in the context of traditional antifungal drugs, has long been a way of obtaining better antifungal effects. For example, Gupta et al. found that miconazole and fluconazole combined with a low dose of amiodarone has a strongly synergistic fungicidal effect [147]. They also found that amiodarone by itself can produce an antibacterial effect by destroying calcium homeostasis in *Saccharomyces cerevisiae* cells. Thus, it is potentially an effective antibacterial drug that can also bring new vitality to traditional antifungal drugs.

Figure 1A shows the mechanism of some antifungal peptides. Pradimycin, amphotericin B, and fengysin can induce apoptosis through the accumulation of ROS. In addition, pradimycin can also bind to the transmembrane protein sln1 to induce apoptosis. Itulin and C16-FengycinA can not only accumulate ROS to induce oxidative stress, but also damage the cell wall to cause cytoplasmic extravasation to induce osmotic stress. However, the activated HOG-MAPK pathway can resist this oxidative stress and osmotic stress. In addition, iturin can directly affect the activity of pathogens by inhibiting cell wall integrity. AMP-jsa9 can inhibit the synthesis of the cell membrane and cell wall of pathogenic bacteria, destroy the cytoskeleton, and affect the normal life activities of pathogenic bacteria by regulating the expression of related proteins. Figure 1B shows two models of destruction of cell membrane by microbial antifungal peptides. Carpet-like model (syringomycin family of lipopeptides): the attached peptides aggregate and insert into the membrane so that the hydrophobic region is aligned with the lipid, and the hydrophilic region is inward to form pores. Barrel wall model (amphotericin B): the peptide forms a large layer parallel to the membrane surface to destroy the membrane. Figure 1C shows the main membrane structure of pathogenic fungi affected by antimicrobial peptides. C16-FengycinA can reduce the hydrophobicity of pathogen and inhibit the synthesis of glucan and chitin, Amp-jsa9 can destroy the cytoskeleton and reduce the content of chitin and ergosterol, and Aureobasidin can inhibit the synthesis of sphingolipids. The decrease of the content of these substances is related to the downregulation of the expression of related proteins by antimicrobial peptides.

## 6. Expectations

Future research should focus on exploring new antifungal peptide resources and developing further antifungal microorganisms with excellent activity and low toxicity. It will be necessary to screen and purify the antifungal peptides discovered, determine their structure–activity relationships, and find ways to artificially synthesize them. Naturally occurring antifungal peptides can be structurally modified using genetic engineering techniques and bioinformatics, so as to obtain antifungal peptides that are more efficient, stable, and safe. From the point of view of treating fungal infections, the development of drug delivery systems and use of new drug combinations are important directions to explore. The overall aim must be to develop antifungal biological agents that are more suitable for use in clinical prevention and treatment applications.

## Data Availability

Data availability in this paper.
